# UPLC-MS/MS analysis and chemical profile of *Ficus pertusa* L *.* bark, an ethnomedicinal resource from intertropical America.

**DOI:** 10.12688/f1000research.154761.1

**Published:** 2024-09-10

**Authors:** Ricardo D.D.G. de Albuquerque, Mayar L. Ganoza-Yupanqui, Frank Romel León-Vargas, Rosa Souza-Nájar, Daniel Carrasco-Montañez, Yessenia Ramos-Rivas

**Affiliations:** 1 Laboratorio de Control de Calidad, Facultad de Farmacia y Bioquímica, Universidad Nacional de Trujillo, Trujillo, La Libertad, 13001, Peru; 2 Departamento de Ingeniería Química, Facultad de Ingeniería Química, Universidad Nacional de la Amazonia Peruana, Iquitos, Loreto, Peru; 3 Departamento de Quimica, Facultad de Ingeniería Química, Universidad Nacional de la Amazonía Peruana, Iquitos, Peru; 4 Departamento de Ciencias Clínicas, Facultad de Medicina, Universidad Nacional de la Amazonia Peruana, Iquitos, Peru

**Keywords:** Natural resource, phenolic compounds, .catechin, afzelechin, chlorogenic acid

## Abstract

**Background:**

*Ficus pertusa* is a native species from intertropical America used for medicinal purpouses. However, despite its importance as ethnobotanical resource, there is poor chemical information about its extracts. Therefore, the objective of this study was to evaluate the phytochemical profile of the hydroalcoholic extract of *F. pertusa* barks through UPLC-MS/MS, as well as its phenolic and flavonoid content.

**Methodology:**

The chemical identification was performed by Ultra-High Liquid Chromatography coupled to Mass Spectrometry, whereas the Total Phenolic content and Total Flavonoid quantification were measured through UV spectrometry.

**Results:**

As a result, 19 substances were identified, which are mostly included within the group of condensed tannins, phenolic acids and flavonoids, such as procyanidins, chlorogenic acid and luteolin.

**Conclusion:**

The identification of the main substances of *F. pertusa*’s bark and the measurement of its polyphenols content can guide the understanding of the pharmacological mechanisms involved in its ethnomedicinal uses.

## Introduction

*Ficus pertusa* commonly known as “renaquilla”, “renaco”, “matapalo”, “yapit”, among others, is a Moraceae native from Mexico, Central and South America and used by local people to treat health problems. It has been reported that the bark is usually oriented in case of inflammations, herniations and traumatisms, whereas other reports mention the use of this species for toothache treatment and as a natural herbicide. ^1^^–^^3^ Furthermore, previous studies shown the anti-inflammatory and analgesic activity of extracts and fractions from *F. pertusa*. ^2^^,^^4^ However, scarce information about its chemical composition is found in the literature, so that until this date, no chemical works were described for the polar compounds from *F. pertusa* bark. Then, the aim of this work was to evaluate the main bark’s phytoconstituents through UPLC-MS/MS analysis, as well as quantify its phenolic profile, which can help to understand the basis for the ethnomedicinal use and its biological and pharmacological mechanisms.

## Methods

### Plant material and preparation of the extracts

*Ficus pertusa* bark was collected from IMET (3.7646°S - 73.2726°W; 3.7643°S - 73.2721°W), Iquitos, Peru, on December 14, 2022. The plant material (1.5 g) were powdered and exhaustively extracted at room temperature with 96% ethanol (100 mL, local production). After evaporation under reduced pressure (40°C), it was obtained the crude hydroethanolic extract (15.5 mg). The identification of the substances were carried out using Waters MassLynx SCN781 version 4.1. MassLynx™ software (Free Trial is disponible). After, Thin Layer Chromatography (TLC) was performed using standard methods for terpenoids, saponins and tannins identification. ^5^

### UPLC-MS/MS chemical identification

A UPLC Waters XEVO TQ-XS system (Register N° 042920-2022 National University of Trujillo – Peru) was employed for the chemical analysis of *F. pertusa.* For this analysis, 2 mg of the dried material was dissolved in 2 mL of methanol and then filtered through a 0.22 μm PTFE filter. Liquid chromatography was conducted using an ACQUITY UHPLC HSS C18 column (100 mm × 2.1 mm, 1.8 μm, Waters) maintained at 25°C. The mobile phases consisted of 1% formic acid (Sigma-Aldrich) aqueous solution (A) and acetonitrile (Sigma-Aldrich) with 1% formic acid (B). The gradient program began with 10% B at the start, increased to 20% B over 6 minutes, then ramped up to 50% B in 1 minute, followed by a rise to 55% B within 7 minutes, further increased to 90% B in 12 minutes, and finally returned to the initial conditions within 4 minutes. The flow rate was set at 0.3 mL per minute, and the injection volume was 10 μL. Detection of all compounds was carried out using a triple quadrupole mass spectrometer equipped with an electrospray ion source (ESI) operating in both positive and negative ionization modes. The cone voltage was maintained at 40 V, with a drying temperature of 450°C and a dry gas flow rate of 13 L/min. Nitrogen was utilized as the dry gas, nebulizing gas, and collision gas. The collision energy was set to 30 eV. HRESIMS and MS/MS spectra were recorded over an m/z range from 50 to 2000 amu.

### Total phenolic content

Total phenol content of *F. pertusa* extract was estimated using Folin-Ciocalteau reagent based assay using gallic acid as standard. ^6^ Each extract was dissolved in distillate water (29.5 mg/mL) and 500 μL was added to the Folin-Ciocalteau reagent (2.5 mL), followed by the addition of 1.5 mL of 20% of Na2CO3. The mixture was incubated at 50°C for 30 min and the absorbance of the developed colour was recorded at 765 nm using UV-vis spectrophotometer. The same procedure was repeated to aliquots of 10-100 ppm aqueous gallic acid solutions used as standard for calibration curve. Total phenolic value was obtained from the regression equations and expressed as mg/g gallic acid equivalent (GAE). The readings were performed in duplicate.

### Total flavonoid content

The total flavonoid content in the *F. pertusa* extract was measured using a modified version of the method by Rolim et al. The equivalent concentration of total flavonoids in rutin was determined spectrophotometrically at 360 nm, using a standard curve of rutin absorbance for comparison. The standard curve included rutin concentrations of 12.5, 25.0, 50.0, 75.0, and 85.0 μg/mL. A solvent mixture of 95% ethanol and 0.02M acetic acid (99:1) was used to prepare all solutions. Each measurement was taken in triplicate.

## Results and discussion

**Figure 1.**
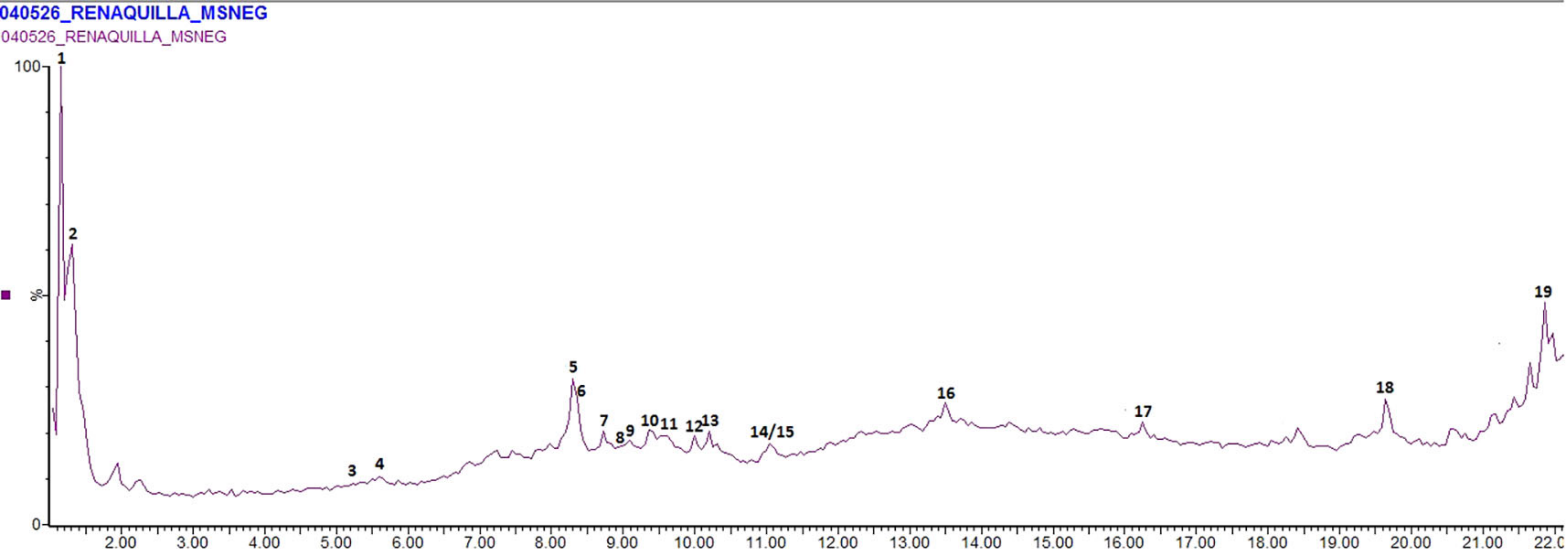
Chromatogram of *F. pertusa* bark extract analysed by UPLC-MS/MS.

In total, 19 substances were identified in the bark extract from *Ficus* sp through UPLC-MS/MS analysis ( Table 1 and Figure 1), so that condensed tannins, phenolic acids and flavonoids were the main secondary metabolites group. The condensed tannins were mostly represented by (epi) catechin and (epi) afzelechin dimers or trimers (presence of monomer fragments m/z = 289 and 273 amu) also known as procyanidins, whereas the phenolic acids protocatechuic acid [M-H = 153]. gentisic acid [m-h = 153], chlorogenic acid [M-H = 353], besides the flavonoids luteolin [M-H = 285] and epicatechin gallate [M-H = 441] were the other substances classified among the phenolic compounds. Also, it was identified the malic acid [M-H = 133] and quinic acid [M-H = 191] as main organic acids from the bark extract, the azelaic acid [M-H = 187] as a representative dicarboxylic acid, and the phenylpropanoid cerberic acid A [M-H = 353]. This profile of metabolites is commonly found for *Ficus* species, so that catechin and afzelechin dimers/trimers-based condensed tannins, as well as their monomers and luteolin derivatives, are prominent substances in these species. ^8^^–^^11^

**Table 1.** Phytochemicals from *Ficus pertusa* bark extract identified by UPLC MS/MS.

N°	Substances	Rt	M-H	Fragments (m/z)
1	Malic acid	1.15	133	115, 73, 71
2	Quinic acid	1.31	191	109, 97, 93, 85
3	Protocatechuic acid	5.20	153	108, 91
4	Gentisic acid	5.63	153	108, 91, 81
5	Catechin	8.30	289	203, 151, 137, 125, 123, 109
6	Chlorogenic acid	8.35	353	191, 173
7	Type B dimer (epi)Catechin-(epi)Afzelechin	8.72	561	289, 123, 109
8	Procyanidin B2	9.03	577	451, 407, 289, 245, 129
9	(epi)Afzelechin-(epi)Catechin-(epi)Catechin	9.15	849	697, 577, 561, 559, 389, 289, 257, 125
10	Type B dimer (epi)Catechin-(epi) Afzelechin II	9.36	561	407, 389, 245, 205, 125
11	(epi)Afzelechin-(epi)Catechin-(epi) Catechin II	9.62	849	582, 563, 543, 431, 407, 299, 291, 289, 161, 121
12	epi-catechin	9.99	289	221, 203, 161, 151, 123, 109
13	epi-afzelechin	10.20	273	161, 135, 97
14	(epi)Afzelechin-(epi)Afzelechin-(epi)Catechin	11.05	833	561, 561, 545, 543, 409, 289, 271
15	Type B dimer (epi)Afzelechin-(epi)Afzelechin	11.05	545	419, 341, 273, 177, 159, 125
16	Epicatechin gallate	13.49	441	397, 378, 330, 202, 161, 139, 113, 101, 89
17	Azelaic Acid	16.27	187	125, 123, 95
18	Luteolin	19.64	285	285, 199, 151, 133
19	Cerberic acid A	21.86	353	297, 283, 201, 151, 107

The flavonoid content expressed in rutin equivalent was equal to 3.14 ± 0.05 %, which was little higher than expected, when compared to the results of quantifying the flavonoids present in different varieties of *Ficus carica*, which in turn showed values within the range between 1.1 and 1.6 % for dry extracts. ^12^ In turn, the total phenolic content expressed in GAE was equal to 213 ± 25 mg GAE/g. The result was also considered high when compared to the aforementioned study, which presented values that covered the range between 42 and 59 mg GAE/g. ^12^

It has been reported that the procyanidins are well known secondary metabolites with broad anti-inflammatory activity, partially explained by their high antioxidant potential, which in turn minimizes the tissue damage in inflammation process. Moreover, procyanidins modulate the arachidonic acid pathway, the inhibition of the gene transcription, protein expression and enzymatic activity of eicosanoid chain, besides supress the production and secretion of inflammatory mediators, the inhibition of mitogen-activated protein kinase (MAPK) pathway activation, and the modulation of NF-ĸB pathway. ^13^^,^^14^ Furthermore, luteolin and chlorogenic acid also can contribute for the anti-inflammatory property of *F. pertusa*, since the first one regulates transcription factors such as STAT3, NF-κB, and AP-1, ^15^ while the chlorogenic acid can decrease the secretion of several pro-inflammatory cytokines, such as IL-8 and IL-6, IL-1β, TNF-α, as well as COX 2 expression. ^16^ A previous chemical study of the *F. pertusa* bark identified the structure of triterpenes and steroids, as for example β-amirin acetate, ^3^ which in turn also possess remarkably anti-inflammatory and analgesic activity, possibly contributing for a synergic effect. ^17^

## Conclusion

In total, 19 substances were identified in the hydroalcoholic extract of *F. pertusa* bark, with a predominance of condensed tannins, phenolic acids, and flavonoids, such as procyanidins, chlorogenic acid, luteolin, among others. The presence of different types of procyanidins and other phenolic compounds may be related to the ethnomedicinal use of the bark, as they are related to anti-inflammatory/analgesic activities. Moreover, the study contributes to the phytochemical knowledge of *F. petusa*, which in turn is a well-described ethnomedicinal resource in native American communities.

## Data Availability

No data are associated with this article.

## References

[1] González-Castañeda N Cornejo-Tenorio G Ibarra-Manríquez G : El género Ficus (Moraceae) en la provincia biogeográfica de la Depresión del Balsas, México. *Bol. Soc. Bot. Mex.* 2010;87:105–124.

[2] Carranza Chavez J : *Evaluación del efecto antiinflamatorio del gel con extracto etanolico de la corteza del ficus pertusa en ratas albinas.* Universidad Inca Garcilaso de La Vega;2018;78. Bachelor’s Thesis.

[3] Mostacero Castro GI : *Caracterización química de triterpenos y/o esteroides obtenidos de la corteza de Ficus pertusa.* Universidad Inca Garcilaso de La Vega;2019;87. Bachelor’s Thesis.

[4] Medina-Marivel H : *Efecto analgésico del extracto etanólico y fracciones de acetato de etilo y acetona de la corteza ficus pertusa en ratones albinos.* Universidad Inca Garcilaso de La Vega;2018;72. Bachelor’s Thesis.

[5] Oliveira AP Raith M Kuster RM : Metabolite profling of the leaves of the brazilian folk medicine *Sideroxylon obtusifolium.* *Planta Med.* 2012;78:703–710. 10.1055/s-0031-1298269 22322398

[6] Valizadeh H Sonboli A Kordi FM : Cytotoxicity, antioxidant activity and phenolic content of eight fern species, from north of Iran. *Pharm. Sci.* 2016;21(1):18–24.

[7] Rolim A Maciel CP Kaneko TM : Validation assay for total flavonoids, as rutin equivalents, from *Trichilia catigua* Adr. Juss (Meliaceae) and *Ptychopetalum olacoides* Bentham (Olacaceae) commercial extract. *J. AOAC Int.* 2005;88(4):1015–1019. 10.1093/jaoac/88.4.1015 16152916

[8] Omar MH Mullen W Crozier A : Identification of proanthocyanidin dimers and trimers, flavone C-glycosides, and antioxidants in *Ficus deltoidea*, a Malaysian herbal tea. *J. Agric. Food Chem.* 2011;59(4):1363–1369. 10.1021/jf1032729 21261251

[9] Elhawary SS Younis IY El Bishbishy MH : LC–MS/MS-based chemometric analysis of phytochemical diversity in 13 *Ficus* spp.(Moraceae): Correlation to their in vitro antimicrobial and in silico quorum sensing inhibitory activities. *Ind. Crop. Prod.* 2018;126:261–271. 10.1016/j.indcrop.2018.10.017

[10] Chen C Peng X Chen J : Antioxidant, antifungal activities of ethnobotanical *Ficus hirta* Vahl. and analysis of main constituents by HPLC-MS. *Biomed.* 2020;8(1):15. 10.3390/biomedicines8010015 PMC7168232 31952281

[11] Afzan A Marcourt L Abd Karim HA : Chemical characterizations dataset of flavonoid glycoside isomers and other constituents from *Ficus deltoidea* Jack. *Data Brief.* 2024;54:110414. 38690315 10.1016/j.dib.2024.110414 PMC11058097

[12] Mahmoudi S Khali M Benkhaled A : Phenolic and flavonoid contents, antioxidant and antimicrobial activities of leaf extracts from ten Algerian *Ficus carica* L. varieties. *Asian Pac. J. Trop. Biomed.* 2016;6(3):239–245. 10.1016/j.apjtb.2015.12.010

[13] Martinez-Micaelo N González-Abuín N Ardevol A : Procyanidins and inflammation: Molecular targets and health implications. *Biofact.* 2012;38(4):257–265. 10.1002/biof.1019 22505223

[14] Valencia-Hernandez LJ Wong-Paz JE Ascacio-Valdés JA : Procyanidins: From agro-industrial waste to food as bioactive molecules. *Foods.* 2021;10(12):3152. 10.3390/foods10123152 34945704 PMC8701411

[15] Aziz N Kim MY Cho JY : Anti-inflammatory effects of luteolin: A review of in vitro, in vivo, and in silico studies. *J. Ethnopharmacol.* 2018;225:342–358. 10.1016/j.jep.2018.05.019 29801717

[16] Liang N Kitts DD : Role of chlorogenic acids in controlling oxidative and inflammatory stress conditions. *Nutrients.* 2015;8(1):16. 10.3390/nu8010016 26712785 PMC4728630

[17] Aragao GF Pinheiro MCC Bandeira PN : Analgesic and anti-inflammatory activities of the isomeric mixture of alpha-and beta-amyrin from *Protium heptaphyllum* (Aubl.) march. *J. Herb.Pharmacother.* 2008;7(2):31–47. 10.1080/J157v07n02_03 18285306

