# Supplementary material for: UPLC-MS/MS analysis and chemical profile of Ficus pertusa L . bark, an ethnomedicinal resource from intertropical America
Source: F1000Res. 2024 Sep 10;13:1042. [Version 1] doi: 10.12688/f1000research.154761.1 (PMC12754363; doi:10.12688/f1000research.154761.1)
Supplement: Peer review history

# Reviewer report: Reviewer response for version 1

**DOI:** 10.5256/f1000research.169829.r437921

**Published:** 2025-12-30

**Authors:** Maité Rodríguez-Díaz

**Affiliations:** 1 Metropolitan Technological University, Santiago, Santiago Metropolitan Region, Chile

Dear Editor:

 This study provides an initial phytochemical characterization of the hydroalcoholic bark extract of *Ficus pertusa*, a medicinal species widely used in intertropical America. Using UPLC-MS/MS, the authors report the identification of 19 compounds—primarily condensed tannins, phenolic acids, and flavonoids—and quantify total phenolics and flavonoids by spectrophotometric assays. While the work addresses a clear knowledge gap regarding the polar constituents of *F. pertusa*, several methodological and interpretive aspects require stronger articulation to enhance scientific rigor and impact.

 The species is ethnomedically relevant, and chemical data—especially regarding polar metabolites—are indeed scarce. The identification of catechin/afzelechin oligomers, chlorogenic acid, and luteolin contributes useful baseline phytochemical knowledge. UPLC-MS/MS is a suitable technique for profiling complex phenolic mixtures, and the authors present a reasonably detailed description of chromatographic and MS conditions.

 Limitations: Total phenolic and flavonoid contents are presented without reporting linearity (R² values), limit of detection/quantification, or variability beyond “duplicate” or “triplicate” measurements.

 The extract concentration used for assays seems extremely high (29.5 mg/mL), which may cause saturation or matrix interference in colorimetric tests.

 However, UPLC-MS/MS data presentation is minimal. Only a chromatogram and a short compound list are provided. No MS spectra, fragmentation pathways, retention times, or identification confidence levels (e.g., according to Metabolomics Standards Initiative) are shown. This limits reproducibility and verification by other researchers.

 Although the discussion briefly mentions that catechins and luteolin derivatives are common in Ficus, the analysis would benefit from a more systematic comparison with existing phytochemical profiles of closely related species (e.g., *F. carica, F. benjamina, F. insipida*).

Is the work clearly and accurately presented and does it cite the current literature?

Yes

If applicable, is the statistical analysis and its interpretation appropriate?

Partly

Are all the source data underlying the results available to ensure full reproducibility?

No

Is the study design appropriate and is the work technically sound?

Yes

Are the conclusions drawn adequately supported by the results?

Yes

Are sufficient details of methods and analysis provided to allow replication by others?

Partly

Reviewer Expertise:

Phytochemistry, natural products characterization, ethnopharmacological evaluation of plants extracts

I confirm that I have read this submission and believe that I have an appropriate level of expertise to confirm that it is of an acceptable scientific standard.

## References

[1] : An Overview of Phytochemical and Biological Activities: Ficus deltoidea Jack and Other Ficus spp. *Journal of Pharmacy and Bioallied Sciences* .2021;13(1) : 10.4103/jpbs.JPBS_232_19 11-25 10.4103/jpbs.JPBS_232_19 PMC814291934084044

# Reviewer report: Reviewer response for version 1

**DOI:** 10.5256/f1000research.169829.r437924

**Published:** 2025-12-30

**Authors:** Patrícia Landazuri

**Affiliations:** 1 Universidad del Quindío, Quindío, Colombia

This work presents the chemical analysis and phytochemical profile of the bark of *Ficus pertusa*, an ethnomedicinal resource from intertropical America. *F. pertusa *is a native species used in traditional medicine to treat inflammation, hernias, and toothaches. Despite its use, there is little information on its chemical composition, which motivated this study to evaluate the phytochemical profile of the hydroalcoholic extract of its bark using UPLC-MS/MS.

 Methodology

 The bark was collected in Iquitos, .

 It would be helpful to know if a herbarium classification was performed to confirm that it is indeed the plant being identified.

 In general terms, the work is clear, well-written, of high technical quality, with appropriate methodology, and the results follow from the methodology and can be replicated by others. As indicated before, identification by a herbarium is necessary.

Is the work clearly and accurately presented and does it cite the current literature?

Yes

If applicable, is the statistical analysis and its interpretation appropriate?

Not applicable

Are all the source data underlying the results available to ensure full reproducibility?

Yes

Is the study design appropriate and is the work technically sound?

Yes

Are the conclusions drawn adequately supported by the results?

Partly

Are sufficient details of methods and analysis provided to allow replication by others?

Yes

Reviewer Expertise:

Obtaining extracts from medicinal plants; biochemistry and metabolic pathways, molecular biology

I confirm that I have read this submission and believe that I have an appropriate level of expertise to confirm that it is of an acceptable scientific standard.
